# Ethylene and Sulfur Coordinately Modulate the Antioxidant System and ABA Accumulation in Mustard Plants under Salt Stress

**DOI:** 10.3390/plants10010180

**Published:** 2021-01-19

**Authors:** Mehar Fatma, Noushina Iqbal, Harsha Gautam, Zebus Sehar, Adriano Sofo, Ilaria D’Ippolito, Nafees A. Khan

**Affiliations:** 1 Plant Physiology and Biochemistry Laboratory, Department of Botany, Aligarh Muslim University, Aligarh 202002, India; meharfatma30@gmail.com (M.F.); harshagautam99@gmail.com (H.G.); seharzebus5779@gmail.com (Z.S.); 2 Department of Botany, Jamia Hamdard, New Delhi 110062, India; naushina.iqbal@gmail.com; 3 Department of European and Mediterranean Cultures: Architecture, Environment and Cultural Heritage (DiCEM), University of Basilicata, Via Lanera, 20, 75100 Matera, Italy; dippolito.ilaria@libero.it

**Keywords:** antioxidant, ascorbate-glutathione, ethylene, photosynthesis, salt stress, sulfur

## Abstract

This study explored the interactive effect of ethephon (2-chloroethyl phosphonic acid; an ethylene source) and sulfur (S) in regulating the antioxidant system and ABA content and in maintaining stomatal responses, chloroplast structure, and photosynthetic performance of mustard plants (*Brassica juncea* L. Czern.) grown under 100 mM NaCl stress. The treatment of ethephon (200 µL L^−1^) and S (200 mg S kg^−1^ soil) together markedly improved the activity of enzymatic and non-enzymatic components of the ascorbate-glutathione (AsA-GSH) cycle, resulting in declined oxidative stress through lesser content of sodium (Na^+^) ion and hydrogen peroxide (H_2_O_2_) in salt-stressed plants_._ These changes promoted the development of chloroplast thylakoids and photosynthetic performance under salt stress. Ethephon + S also reduced abscisic acid (ABA) accumulation in guard cell, leading to maximal stomatal conductance under salt stress. The inhibition of ethylene action by norbornadiene (NBD) in salt- plus non-stressed treated plants increased ABA and H_2_O_2_ contents, and reduced stomatal opening, suggesting the involvement of ethephon and S in regulating stomatal conductance. These findings suggest that ethephon and S modulate antioxidant system and ABA accumulation in guard cells, controlling stomatal conductance, and the structure and efficiency of the photosynthetic apparatus in plants under salt stress.

## 1. Introduction

Every year, 1.5 million hectares of lands are becoming inappropriate for agricultural production due to salinity [1,2]. Salinity is one of the most deleterious among abiotic stresses, causing ion imbalance, nutrient deficiency, and oxidative stress [3]. The high content of Na^+^ and Cl^−^ disturbs efficient stomatal regulation together with the reduction in photosynthesis due to the over-production of reactive oxygen species (ROS) [4,5,6]. Stomatal closure has also been associated with membrane damage and disturbs the activity of various enzymes, especially those involved in ATP synthesis and photosynthesis [7]. Under salt stress, plants regulate the thylakoid membrane fluidity and membrane lipid composition by involving various mechanisms in a way to maintain a suitable environment for the functioning of integral proteins [8]. In such efforts, various salt-responsive genes are synthesized that encode for protein mainly involved in the scavenging of ROS, organization of thylakoid membrane, the activity of pigment system II (PSII), assimilation of carbon dioxide (CO_2_), biosynthesis and signaling of abscisic acid (ABA), together with osmotic and ionic homeostasis [9]. The enzymes of ascorbate-glutathione (AsA-GSH) cycle that assist in ROS detoxification are also present within the chloroplast stroma. Thus, it is essential to maintain the thylakoid structure and stomatal regulation together with the antioxidants involved in the AsA-GSH cycle to scavenge ROS, so and alleviating the deleterious effects of salt stress. Akyol et al. [10] reported that antioxidant metabolism helps the plant to deal with salinity-induced oxidative stress.

The exogenous application of substances or signaling molecules can regulate the plant’s metabolism for stress tolerance. Studies have shown that sulfur (S) and ethylene regulate salt tolerance in plants [11,12] by regulating various cellular processes. Sulfur is a major constituent of many enzymes of the photosynthetic carbon reduction cycle, and S supplementation increases photosynthesis via modulating the photosynthesis machinery and activating the synthesis of antioxidants [13,14]. Indeed, S-containing metabolites are reported to change physiological and molecular processes under salinity stress in plants [15]. Lou et al. [16] also reported that S plays an important role in regulating the AsA-GSH cycle for detoxification against cadmium stress.

Ethylene is a gaseous plant hormone that interacts with nutrient uptake and controls plant responses under growth-limiting conditions or stress [17,18,19]. Jiang et al. [20] reported in Arabidopsis that the *Ctr1* mutants retained higher K^+^ and lower Na^+^ concentration in contrast to *ein2* or *ein3* plants and exhibited lesser reduction in leaf area and root elongation compared to *ein2-5* or *ein3-1* mutants. Ethylene interacts with ABA and activates the signaling pathways network to influence the phytohormones regulation of several processes [21]. Ethylene works antagonistically with ABA [22] and is responsible for regulating guard cells signaling [23]. Cao et al. [24] reported that ABA-induced *ERF8* acted as transcriptional repressor of ABA signaling, and *ERF8* knockdown line displayed enhanced ABA sensitivity while overexpression lines showed decreased sensitivity. Ethylene production and S-assimilation had close margins with some common regulatory elements or metabolites [25] and S regulates abiotic stress tolerance via ethylene [26]. Ethylene increases the assimilation of nitrates and sulfates to reduce salinity-induced oxidative stress [12]. The influence of combined ethephon and S application on ABA content and their coordination in stomatal regulation has been only partially reported in other species, such as tomato [27,28], while this information could provide a new insight for controlling photosynthesis under salt stress.

On this basis, we studied ethephon and S-mediated protection of photosynthetic machinery of mustard plants (*Brassica juncea* L. Czern & Coss. var. Varuna) and the involvement of the antioxidant system and ABA under salt stress. This species was selected for its several uses in land restoration, phytoremediation, and food and raw materials production, as well for its genome similarity to other Brassicaceae, such as the model plant *Arabidopsis thaliana*. The functional hypothesis was tested by studying the content of Na^+^, Cl^−,^ and H_2_O_2_, and lipid peroxidation, the structure and function of photosynthetic apparatus, and photosynthetic parameters. The scanning and transmission electron microscopy of stomata were also performed to support the involvement of ethephon and S in the regulation of stomatal behavior. ABA content was studied with ethephon + S to show their coordinating role in regulating ABA content. Norbornadiene (NBD), an ethylene action inhibitor, was applied on ethephon- and S-treated plants under NaCl stress. Finally, ABA and H_2_O_2_ contents, and photosynthesis were studied in detail.

## 2. Results

### 2.1. Effect of Ethephon and Sulfur on Ions Accumulation

Salt stress increased the content of Na^+^ and Cl^−^ ions both in root and leaves (Table 1). Ethephon + S were equally effective in lowering root and leaf Na^+^ and Cl^−^ content under no stress while under salt stress S proved to be better. Root Na^+^ content was reduced by 16.3 and 17.4% and root Cl^−^ by 28.5 and 32.4%, leaf Na^+^ content was reduced by 17.8 and 21.4%, and leaf Cl^−^ content by 23.5 and 26.5% with ethylene + S, respectively, compared to control plants. However, the maximal decrease in the accumulation of Na^+^ and Cl^−^ content was noted when plants received supplementation of both ethephon and S under stress and no-stress conditions.

### 2.2. Ethephon and Sulfur Coordinately Optimize Antioxidant System and Redox State and Reduce Oxidative Stress

The content of H_2_O_2_ and lipid peroxidation were measured to observe the involvement of oxidative stress (Table 2). The individual application of ethephon + S reduced H_2_O_2_ content in plants by 45.9 and 49.6%, respectively compared to control under no stress, but the reductions were about 18.0 and 27.9% under salt stress, compared to the control plants. Similarly, the level of lipid peroxidation in terms of TBARS content increased with 100 mM NaCl compared to the control plants. The observed values for TBARS content in plants receiving ethephon + S without salt showed that both reduced lipid peroxidation by 35.7 and 40.4%, respectively compared to control plants. The utmost reduction in content of H_2_O_2_ and TBARS was found with the combined treatment of ethephon + S under no stress and salt stress, compared to control plants.

The enzymes of the AsA-GSH cycle were activated by the treatment of 100 mM NaCl. The activity of ascorbate peroxidase (APX), glutathione reductase (GR), dehydroascorbate reductase (DHAR), and monodehydroascorbate reductase (MDHAR) increased after the supply of ethephon or S, but to higher extent in ethephon + S. However, under salt stress, the enhancements of 73.7, 52.4, 49.8, and 101.7% in the activity of APX, GR, DHAR, and MDHAR, respectively, with ethephon-treated plants, while the activity of these enzymes increased by 99.1, 67.1 63.1, and 121.7%, respectively, with S, compared to the control (Table 2). The application of combined effect of ethephon + S under salt-treated plants maximally increased the activity of antioxidant enzymes, showing about a two-fold increase in GR and DHAR, and about two and half -fold in APX activity, while MDHAR activity increased by about three times, compared to control plants (Table 2).

To assess the importance of antioxidant metabolism, ethephon and S act as a precise mechanism for the alleviation of salt-induced toxicity. The application of ethephon + S significantly increased the content of GSH, and the redox state (Table 2), AsA and DHA compared to the control (Table 3). Plants grown with ethephon or S increased the content of GSH by 68.9 and 74.1%, redox state by 50.2 and 55.5% (Table 2), and AsA by 68.6 and 71.5% (Table 3), respectively, compared to control plants. Similarly, DHA also increased about three folds by ethephon or S alone. The maximal increase in these characteristics was obtained by the combined treatment of ethephon + S under no stress and stress conditions than their individual application. The supplementation of S was more effective in increasing content of GSH, AsA, and DHA and redox state than individual application of ethylene. Under salt stress, S acted better than ethylene in alleviating salt stress effects, probably because of higher S-assimilation and the antioxidant enzymes scavenging capacity. However, applied ethephon and S together helped in the better utilization of excess S (200 mg kg^−1^ soil) for higher GSH synthesis and, consequently, caused a reduction in oxidative stress, with lower H_2_O_2_ and TBARS content.

### 2.3. Ethephon and Sulfur Regulate ATP-S Activity and S Content

Under salt stress, ATP-S activity and S content increased which were further increased by ethephon + S (Table 3). Both were equally efficient in enhancing S-assimilation under no stress, while under salt stress S more efficiently enhanced ATP-S activity and S content (Table 3). The highest increase in S-assimilation was observed with the combined application of ethephon + S under no-stress and salt stress conditions. The demand for increased thiols for removal of excess ROS under salt stress was met with higher S-assimilation by ethylene when excess-S was present, through increased activity of ATP-S, resulting in an efficient detoxification of ROS.

### 2.4. Effects of Ethephon and Sulfur on ACS Activity and Ethylene Emission

**Figure 1:**
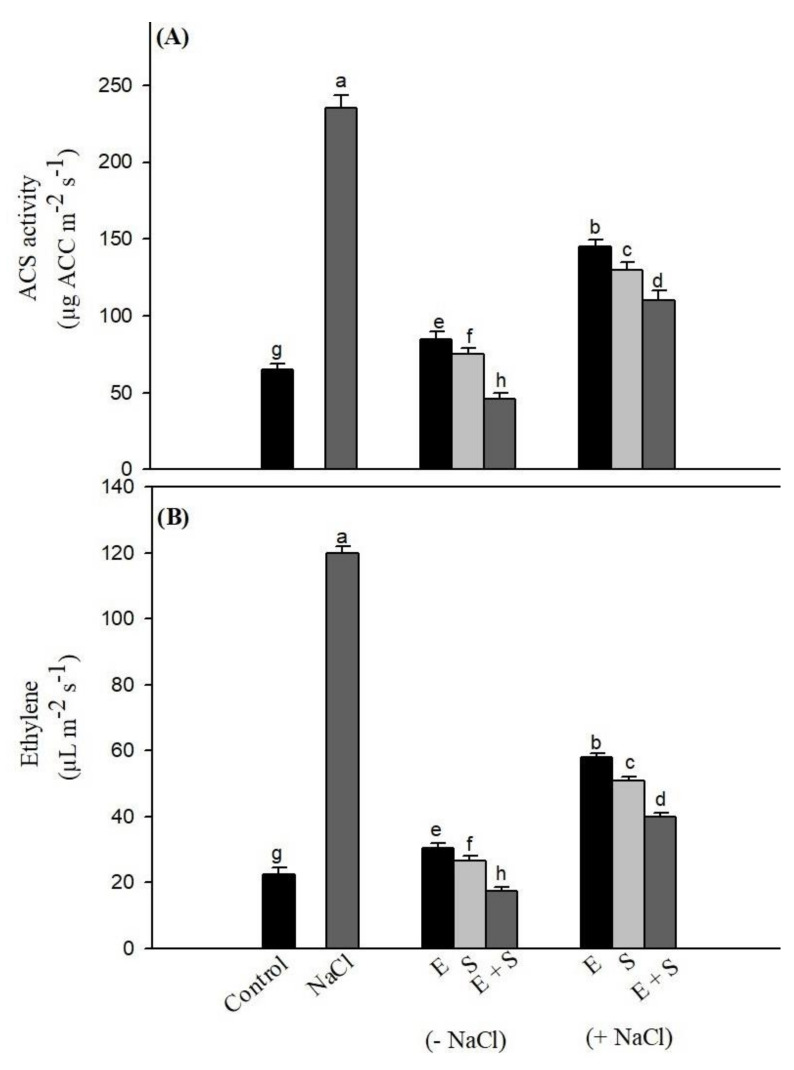
(**A**) Activity of 1-aminocyclopropane carboxylic acid synthase (ACS) and (**B**) ethylene emission in mustard leaves at 30 days after sowing treated with 200 µL L^−1^ ethephon (E) and/or 200 mg S kg^−1^ soil (S) in presence or absence of 100 mM NaCl. Data are presented as treatments mean ± SE (*n* = 4). Data followed by same letter are not significantly different by LSD test at *p* < 0.05.

Plants subjected to salt stress enhanced ACS activity and ethylene emission with several-fold (Figure 1). The application of ethephon and/or S was efficient in increasing ACS activity and ethylene under no-stress conditions. However, under salt stress, both ACS activity and ethylene were considerably reduced by both ethephon and S. The maximum reduction in ethylene emission was observed with combined ethephon + S treatment, that brought ethylene to a favorable level to enhance photosynthesis and growth by increasing the synthesis of AsA, GSH, together with other enzymes of the AsA-GSH cycle.

### 2.5. Effects of Ethylene and Sulfur on Stomatal Response and Chloroplast Ultrastructure

**Figure 2:**
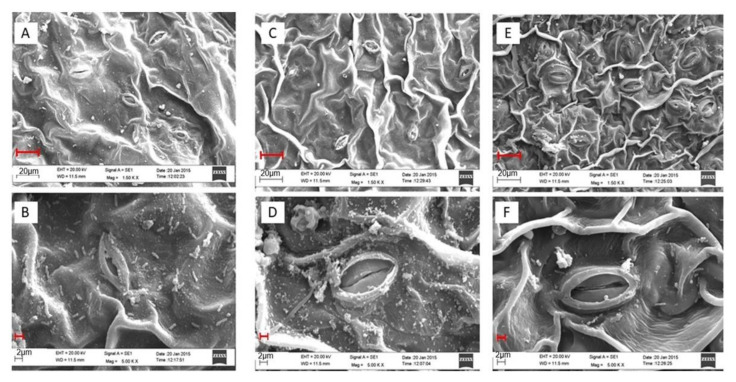
Stomatal behavior of mustard leaves (**A**,**B**) under control, (**C**,**D**) 100 mM NaCl, and (**E**,**F**) 200 mg S kg^−1^ soil + 200 µL L^−1^ ethephon with 100 mM NaCl at 30 days after sowing. The opening and closing of stomata were observed under scanning electron microscope at a magnification of 1.5 kx (**A**,**C**,**E**) and 5.0 kx (**B**,**D**,**F**) in mustard leaves treated with 100 mM NaCl at 30 d after sowing. Bars (**A**,**C**,**E**) = 20 µm; bars (**B**,**D**,**F**) = 2 µm.

The image of the stomatal aperture was apparently visible under different treatments when SEM was performed. Leaf samples under control conditions had normal stomata with the characteristic guard cells Figure 2A,B. The impact of salt stress on stomatal closure is clearly seen in Figure 2C,D where the stomatal density was reduced by 19.0% under salt stress, as compared to the control plants. However, the combination of ethephon + S resulted in reduced effects of salt stress in plants and stomatal response was partially recovered with comparatively opened stomatal aperture (Figure 2E,F).

**Figure 3:**
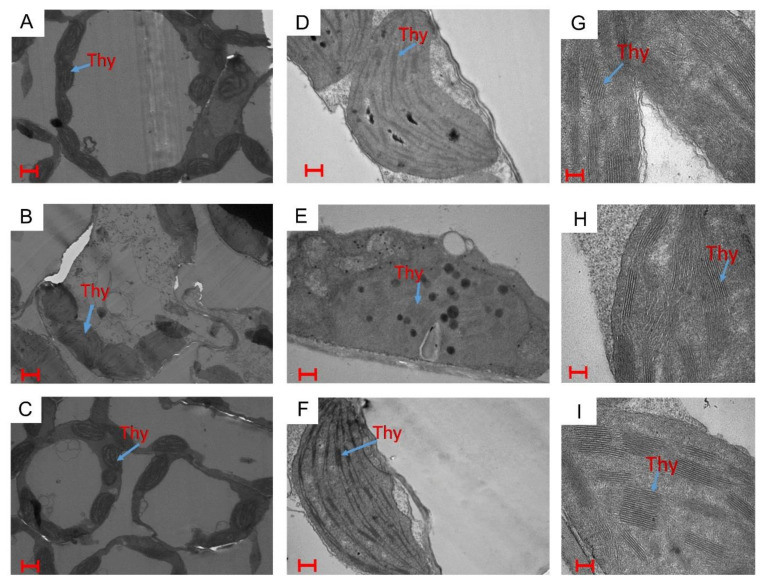
Ultrastructure of chloroplasts in mustard leaves using transmission electron microscopy at a magnification of 1.2 kx (**A**–**C**), 6.0 kx (**D**–**F**) and 15.0 kx (**G**–**I**) under (**A**,**D**,**G**) control, (BEH) 100 mM NaCl, and (**C**,**F**,**I**) 200 mg S kg^−1^ soil + 200 µl L^−1^ ethephon with 100 mM NaCl at 30 d after sowing. Bars (**A**–**C**) = 500 nm; bars (**D**–**F**) = 100 nm; bars (**G**–**I**) = 20 nm. Thy = thylakoid membranes.

The ultrastructure of chloroplasts as seen under transmission electron microscopy micrographs (Figure 3) showed the influence of different treatment on the chloroplast structure. Under normal conditions, chloroplasts had a regular shape with well-arranged thylakoid systems (Figure 3A,D,G). There were disturbances in chloroplast ultrastructure under NaCl treatment and disorganized thylakoid systems were observed with a significant increase in size (Figure 3B), that was clearer in Figure 3E,H. However, the treatment with 100 mM NaCl plants with ethephon + S showed a marked modification in chloroplast ultrastructure. Here, chloroplast had a regular shape with well-arranged thylakoid systems and contained a markedly increased number of thylakoid stacks, as evident in Figure 3C,F,I.

**Figure 4:**
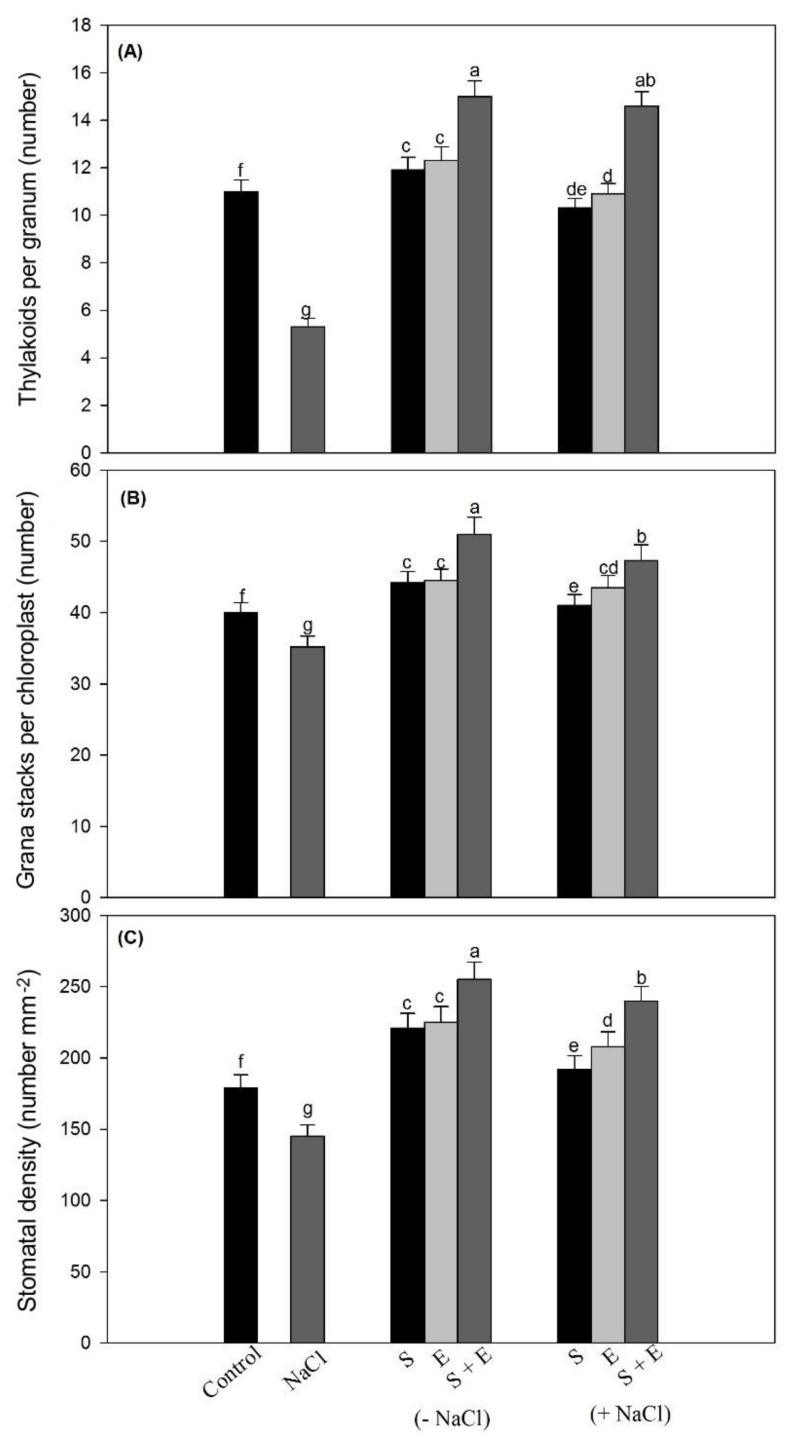
Number of thylakoids per granum (**A**), number of grana stacks per chloroplast (**B**) and stomatal density (**C**) in mustard leaves at 30 days after sowing treated with 200 µL L^−1^ ethephon (E) and/or 200 mg S kg^−1^ soil (S) in presence or absence of 100 mM NaCl. Data are presented as treatments mean ± SE (*n* = 4). Data followed by same letter are not significantly different by LSD test at *p* < 0.05.

The number of thylakoids per granum decreased by 51.8% under salt stress, while a maximum increase of 32.7% in thylakoids per granum was seen with the combined ethephon + S treatment under salt stress (Figure 4). The number of grana stacks per chloroplast decreased by 12.0% under salt stress, while it was increased by 18.2% with the combined ethephon + S treatment under salt stress (Figure 4). The combined ethephon + S treatment increased stomatal density by 34.1%, compared to control (Figure 4).

### 2.6. Supplementation of Ethephon and Sulfur Coordinately Increases Photosynthetic Efficiency, Pigment Content and Growth Parameters

Substantial reductions in chlorophyll (Chl) and anthocyanin content, PSII activity, WUE, and gas exchange parameters were noted with 100 mM NaCl, compared to the control (Table 4 and Table 5). Furthermore, photosynthetic parameters and pigment content were increased by both ethephon and S treatment, compared to the non-stressed control plants (Table 4 and Table 5). Chl a, Chl b, total Chl, carotenoids and anthocyanin content were studied to investigate about the role of ethephon and S on photosynthesis. These photosynthetic pigments were reduced under salt stress except anthocyanins, that increased under salt stress and further increased with ethephon and/or S treatments. Chl a, Chl b, total Chl, and carotenoids declined by 16.7, 33.8, 22.0, and 19.2%, respectively, compared to the control plants (Table 4). Both ethephon and S equally increased the studied photosynthetic pigments, but ethephon + S combined application caused an increase of Chl a by 29.8%, Chl b by 51.6%, total Chl by 32.0%, and carotenoids by 36.8%. Anthocyanin content increased under salt stress, and ethephon + S application caused a further three-fold increase in anthocyanins (Table 4).

The treatment with ethephon increased PSII efficiency by 15.3%, stomatal conductance by 27.5%, intercellular CO_2_ concentration by 38.9%, net photosynthesis by 40.9% and WUE by 32.3%, compared to control plants, whereas the treatment with S increased PSII activity by 16.6%, stomatal conductance by 29.1%, intercellular CO_2_ concentration by 42%, photosynthesis by 43.1%, and WUE by 34.7% compared to the control (Table 5). The maximal increase in gas exchange and photosynthetic efficiency was noted with ethephon + S, a treatment that improved PSII activity by 26.9%, stomatal conductance by 56.5%, intercellular CO_2_ concentration by 68.5%, photosynthesis by 62.4%**,** and WUE by 61.7% compared to control (Table 5). Under salt stress, the combined ethephon + S increased photosynthetic parameters more than the individual application of ethephon or S, but these increases were lower than those occurring without stress conditions.

The growth characteristic, leaf area, and plant fresh weight decreased significantly under salt stress, while ethephon + S increased these characteristics, both in absence or presence of NaCl, compared to the control (Table 5). Plants grown with ethephon or S individually increased the leaf area by 40.8 and 44.1%, and plant fresh weight by 36.7 and 39.5%, respectively, compared to the control. In the presence of salt, the decrease in leaf area and plant fresh weight was maximally overcome with ethephon + S (Table 5). Plants grown with salt and treated with ethephon or S exhibited an increase of leaf area by 18.5 and 33.0%, and of plant fresh weight by 19.0 and 29.3%, respectively, compared to the control plants (Table 5).

### 2.7. Effects of Norbornadiene on ABA/H_2_O_2_ Content, Stomatal Regulation, and Gas Exchange

**Figure 5:**
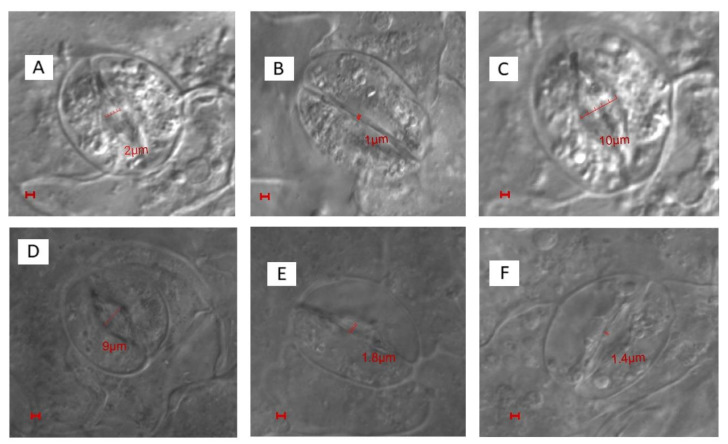
Stomatal behavior of mustard leaves (**A**) under control, (**B**) 100 mM NaCl, (**C**) 200 mg S kg^−1^ soil + 200 µL L^−1^ ethephon without NaCl, (**D**) 200 mg S kg^−1^ soil + 200 µL L^−1^ ethephon with NaCl, (**E**) 100 μM norbornadiene (NBD), and (**F**) 100 μM norbornadiene (NBD) in presence of 100 mM NaCl with 200 mg S kg^−1^ soil + 200 µl L^−1^ ethephon at 30 days after sowing. The stomatal opening and closing response was studied using confocal microscopy. Bars = 1 µm.

The supplementation of norbornadiene (NBD; an ethylene action inhibitor) alone and in combination with ethephon + S in the presence of NaCl resulted in the increase in H_2_O_2_ and ABA content, with subsequent decreases in net photosynthesis and stomatal conductance, and inhibition of stomatal response and stomatal opening, compared to the plants treated with ethephon + S under NaCl stress and compared to the control (Table 6 and Figure 5). This confirms that combined application of ethephon and S under salt stress alleviates the ABA-mediated stomatal closure and increase stomatal aperture width and conductance. This, in turn, led to an increase in photosynthesis under salt stress via ethylene action on guard cell, that also lowered H_2_O_2_ content. (Table 2).

The application of ethephon + S decreased ABA content by 59.6% and H_2_O_2_ content by 74.0% compared to NaCl treatment under salt stress. It increased stomatal conductance and photosynthesis under salt stress compared with the control. The application of NBD to ethephon + S treatment under NaCl stress increased both ABA and H_2_O_2_ content, resulting in decreased stomatal closure and photosynthesis, highlighting the role of ethylene in inhibiting ABA-mediated stomatal closure (Table 6). The results showed that the treatment of 100 mM NaCl led to a reduction of the stomatal aperture width, compared to the control (Figure 5). Ethephon + S application to plants receiving NaCl showed increased stomatal width by five times, compared to the control, and by 9.5 times compare to the salt treatment (Figure 5). NBD treatment reduced stomatal width either alone or in combined ethephon + S in plants under salt stress.

To strengthen the mechanisms of stomatal opening, confocal microscopy was used with ethephon + S, either alone or in combination with stress. The results showed that the salt-treated plants have stomata with the marginal opening of 1 µm in diameter (Figure 5B), while it was 2 µm in control plants (Figure 5A). However, the most prominent effect on stomatal opening was observed in plants treated with ethephon + S, with a value of 10 µm (Figure 5C). These plants showed the largest opening of the stomata followed by 9 µm in diameter of stomatal width for ethephon + S with salt, compared to control and salt-treated plants (Figure 5D). The application of NBD to the plants treated with ethephon + S and NaCl decreased the diameter of stomatal aperture width to 1.4 µm (Figure 5F).

## 3. Discussion

The present study suggested a tight correlation between ethephon and S in plants for alleviating salt stress, and as the most positive effects were found with the combination of ethephon with S. This treatment resulted in higher AsA content, low DHA content, with high DHAR activity, compared to the other treatments (Table 2 and Table 3). Higher GR activity helped to maintain the reduced GSH level and higher redox state (GSH/GSSG) (Table 2). The DHAR enzyme uses GSH as the substrate to reduce DHA to AsA, and GSH is oxidized to GSSG [29,30,31,32,33,34,35,36]. In our experiment, it was GR that functions here to reduce the generated GSSG back to its reduced form (GSH) by NADPH dependent reaction [37]. Singh et al. [38] reported that under arsenic stress, S and Ca efficiently regulated the AsA-GSH cycle to maintain the redox state. The study of Asgher et al. [39] showed that ethylene potentially reduced Cd stress in mustard by increasing the GSH in the presence of S. Ethephon increased GR activity (Table 2), likey because of the increased overexpression of γ-ECS and GSH synthetase in the chloroplasts. GR enhances the antioxidant enzyme activities and enhances tolerance to oxidative damage under salt stress [40]. Also, ethephon and S treatments maximally enhanced DHAR activity to regenerate AsA and helped in maintaining the redox status of the cell together with the glutathione pool (Table 2). MDHAR has a key role in sustaining a reduced pool of AsA and maintains its redox state together with DHAR under stress conditions [36]. Increased MDHAR activity upon ethephon and S treatment under salt stress (Table 2) suggested the potentiality of this enzyme in reducing salt-induced oxidative stress and increasing reduced AsA pool. Thus, the activation of the AsA-GSH cycle by the coordinated action of ethephon and S under salt stress helped in the reduction of salt-induced oxidative stress (H_2_O_2_ and TBARS) (Table 2). Sauter et al. [41] have shown that the production of S metabolites is an essential link between S-assimilatory enzymes and ethylene biosynthesis. Glutathione acts as a signal, controlling the inter-organ regulation of S nutrition and is mainly confined to the leaves and worked as an important antioxidant and protector of plants under stress and the major non-protein S source in plants [42,43]. S-assimilation can influence ethylene signaling in plants [44,45].

When applied together, ethephon and S acted in coordination to increase S-assimilation through increased ATP-S activity and S-content (Table 3). ATP-S catalyzes SO_4_^2−^ activation and yields adenosine-5′-phosphosulfate (APS), that is reduced to sulfide and incorporated into cysteine (Cys). Cysteine acts as a precursor or donor for various S-containing metabolites, including methionine and GSH. Methionine controls ethylene formation through its first metabolite S-adenosylmethionine. The involvement of ATP-S in plant-tolerance to abiotic stresses occurs via different S-compounds. Moreover, ATP-S is related with ethylene, and this is supported by the role of *EIN3* and *EIL1*, two members of EI3/EIL TF family, as central regulators of ethylene signaling [46]. In the present study, ethephon affected ATP-S activity and S assimilation and enhanced the plants’ tolerance to salt stress (Table 3). Ethephon effects on increasing the S-assimilation and excess S-induced GSH production under salt stress for the alleviation of salt toxicity have been reported [18,44,45].

Ethylene plays a role in regulating synthesis of S compounds and controls plant processes and stress tolerance [19,47]. It has been reported that ethylene interacts with Se and improves the antioxidant system under Cd stress by inducing defense responses [48,49]. Lin et al. [50] demonstrated that application of exogenous ACC to salt pre-treated *Arabidopsis thaliana* Col-0 seeds decreases the concentration of H_2_O_2_ in germinating seeds and improves salt tolerance. Finally, ethylene significantly reduces ROS production and TBARS, and increases GSH levels [51]. Plants grown with ethephon + S not only showed a reduction in ethylene emission (Figure 1) but also an increased ethylene perception, useful to alleviate salt stress via an increase in APX/GR activities and GSH content (Table 2), and photosynthesis (Table 5).

Ethylene-mediated variation in stomatal response [52,53,54] has been shown to influence photosynthesis [47]. Under salt stress, stomatal closing occurred, likely because of the phytotoxic effect of salt. Indeed, to the excessive accumulation of Na^+^ and Cl^−^ (Table 1), the guard cells became flaccid, which resulted in stomatal closing (Figure 2). Hormonal action is yet another cause of this closure, and as an avoidance mechanism, ABA is involved in stomatal closure to avoid water loss [33,34]. However, this compromises the plant photosynthetic potential because of lower stomatal conductance. The application of ethephon + S highly influenced the osmotic relations that resulted in stomatal opening (Figure 2). The regulation of the stomatal aperture (Figure 2) by the guard cells was crucial for minimizing water loss from leaf tissues and maximizing CO_2_ exchange for photosynthesis (Table 5). Similarly, in studies on cucumber and mustard, disorganized thylakoid membranes were observed after the salt treatment, with a high decrease in chlorophyll concentration simultaneously [4,55].

It was reported that under stress conditions, the lipid-to chlorophyll ratio was markedly increased, showing that the protein-packing density in thylakoid decreases under stress [4,56]. However, the treatment of ethephon + S under salt stress showed higher modification in chloroplast ultrastructure (Figure 3). In this treatment, the chloroplasts showed regular shape with well-arranged thylakoid systems and contained a markedly increased number of thylakoid stacks (Figure 3). Combined ethephon + S treatment resulted in lower level of lipid peroxidation (Table 2) and showed higher chlorophyll contents (Table 4) than control or salt-treated plants as shown. Thus, it is possible that the lipid-to-chlorophyll ratio is lower, or the plants have more chloroplasts per cell or more thylakoid membranes per chloroplast than the control or salt-treated plants.

Studies suggest that the increase in efficiency of PSII by ethylene treatment under nickel and Zn, Se, and Cr stress involved an increase in the electron transport that helped plants to limit singlet oxygen production at PSII resulting in increased PSII activity [45,57]. Ethylene had a regulatory interaction with GSH leading to an increase in the reduced cell environment under salt stress (Table 2 and Figure 1). This cumulatively protected chloroplast and enzymes of the Calvin–Benson cycle. The influence of S on photosynthetic characteristics is well recognized as a virtue of stabilization of enzyme protein structure [4]. Ethephon application has been shown to increase photosynthesis through increased use efficiency of nutrients, stomatal conductance and Rubisco activity [45,47]. It has been reported that ethylene signaling is required for salt-tolerance as ethylene insensitive mutant showed more salt sensitivity [58]. Several studies have shown that the ethylene response depends on the availability of mineral nutrients [19,39,45,59]. Iqbal et al. [60] reported the existence of a regulatory interaction between ethylene, proline and N for salt tolerance by effecting N assimilation under variable N level under no salt or salt stress in mustard. The supply of S under Fe-deficient condition has a role in regulating the biosynthesis of ethylene, which is a major component involved in Fe uptake [61]. It has also been shown that ethylene has the potential to control S metabolism under Cd and Cr stress [39,45]. A substantial increase in enzymatic and non-enzymatic antioxidants with ethephon and S under salt stress (Table 2 and Table 3) helped plants in reversing the effects of salt-induced ROS on photosynthesis. Ethephon and S are essential to recover polypeptides from salt-induced down-regulation of protein synthesis. Indeed, it has been reported that ethephon at 50 and 100 ppm increased protein band intensities compared to untreated plants of broad bean [62]. The higher leaf area observed (Table 5) was likely correlated with ethephon-enhanced ethylene biosynthesis (Figure 1), as also found in other studies [45,47].

It is known that ABA increases under stress and handles stomatal closure as a defense mechanism. However, this is the trade-off that restricts photosynthesis and growth in plants because of the limitation in gaseous exchange [63]. Tanaka et al. [64] reported that ABA is synthesized under stress conditions and induces stomatal closure to reduce transpirational water loss. Stomata are the portal for both water exit and CO_2_ diffusion into leaf for photosynthesis. Stomatal regulation for the conservation of water occurs at the expense of CO_2_ diffusion into leaf tissue and subsequent reduction in photosynthesis under drought [32]. Both ethylene and ABA are involved in regulating stomata, and they act in an antagonistic manner [65,66]. Combined ethephon + S reduced H_2_O_2_ content due to higher activity of AsA-GSH cycle enzymes (Table 2) and showed stomatal opening (Figure 2) by limiting ABA production (Table 6) under salt stress. The high content of H_2_O_2_ in guard cells is responsible for stomatal closure, together with the ABA [67]. NADPH oxidases are reported to be a source of ROS in the plasma membrane and are required for stomatal closure. Furthermore, the double mutant *atrbohD*/*F* was found to be impaired partially in stomatal closure induced by ABA [68]. Mutants that are ABA-insensitive exhibited reduced ROS formation in guard cells but normally responded to exogenous H_2_O_2_ supplementation suggestive that the genes responsible for ABA-insensitivity acted upstream of H_2_O_2_ generation [69]. The reduction in ABA also led to enhanced antioxidative enzyme activity. Ethylene signaling mutant (*etr1*, *ein2*, *ein3*) and ABA pathway mutants (*aba1*, *aba2*, *abi1*, *abi2*) are reported to antagonistically regulate the expression of defense and stress-responsive genes under biotic and abiotic stress responses [70,71,72]. It was observed that under no-stress conditions, ethephon + S reduced ABA accumulation, while salt stress increased ABA by 3.5 times compared to the control (Table 6). Supplementation of combined ethephon + S under salt stress decreased ABA level (Table 6) and increased stomatal aperture (Table 4). Beguerisse-Diaz et al. [73] reported that when ABA and ethylene dose are applied together, guard cells exhibited increased antioxidant activity that reduced H_2_O_2_ that prevented stomatal closure suggestive of crosstalk between the two hormones. Similar to the reported study, when ethephon and S were applied to plants receiving ABA, the content of ABA was reduced, and stomatal closure was inhibited. Tanaka et al. [66] suggested that ABA-induced stomatal closure was inhibited by ethylene treatment in *Arabidopsis*.

ABA treatment is reported to enhance H_2_O_2_ production in *Vicia faba* at high air humidity [74]. Ethylene alters the rate of photosynthesis by affecting the diffusion rate of CO_2_ from the atmosphere to the intercellular cavities by influencing stomatal aperture [75]. Kumar et al. [76] suggested the role of ethylene and ABA in inducing stress-responsive genes and proteins by activating GSH biosynthesis to reduce abiotic stress conditions in the plant. ABA is reported to stimulate ROS production under salt stress; however, the relationship between stress responses and plant growth is still not fully understood [77,78,79]. Ethephon + S enhanced the antioxidative system in plants to reduce H_2_O_2_ signaling (Table 2). Ethylene is reported to stimulate flavonol production in the guard cells that results in the reduction of ROS and suppresses ABA-induced stomatal closure [80,81]. The above results can be summarized by the regulatory role of ethylene in salt tolerance via its positive influence on plants S-assimilation, enhancing the activity of AsA-GSH cycle that detoxifies H_2_O_2_ content, reduces oxidative stress, and regulates photosynthesis by inhibiting ABA-induced stomatal closure.

However, the regulation between ethylene and ABA cannot be isolated as a sole criterion for stomatal regulation because hormonal interaction occurs in a cascade of reaction affecting each other responses. While ethylene regulates stomatal conductance by affecting ABA content, ABA interacts with NO, H_2_S, H_2_O_2_ and other molecules, such as phytohormones, for stomatal regulation. Tanaka et al. [64,66] reported that cytokinins and auxins regulate stomatal movement but are antagonistic to ABA action on stomatal movement while they enhance ethylene biosynthesis. This is suggestive that the inhibitory effect of cytokinin and auxin on ABA-induced stomatal closure is via an increase in ethylene and ethylene inhibits the ABA-induced reduction of osmotic pressure in the guard cells, that is responsible for stomatal closing. Also, ethylene may inhibit stomatal closure by enhancing flavonol accumulation, which reduces ROS accumulation in guard cells [80,81] or may induce stomatal closure via ROS production in guard cells [53,82] depending on the experimental conditions. Munemasa et al. [83] reported that both ABA and methyl jasmonate (MeJA) induce stomatal closure while ethylene inhibits MeJA and ABA-induced stomatal closure by reducing ROS production and targeting S-type anion channels. The activation of the S-type anion channels triggers plasma membrane depolarization and K^+^ efflux from guard cells [84]. Efflux of K^+^ reduces guard cell pressure and thus stomatal closure. She and Song [65] found in *Vicia faba* that ABA-induced stomatal closure was mediated via nitric oxide while ethylene was found to inhibit ABA-induced stomatal closure by decreasing the NO production. Besides, H_2_O_2_ and NO molecules engage in crosstalk with Ca^2+^ ions that form a complex signaling network in plants experiencing abiotic stresses [85].

## 4. Materials and Methods

### 4.1. Plant Material, Growth Conditions, and Treatments

Healthy seeds of mustard (*Brassica juncea* L. Czern., var. Varuna) were surface sterilized with 0.01% HgCl_2_, followed by repeated washing with distilled water, and sown in 25-cm deep pots containing soil with peat and compost (4:1, *w*/*w*) mixed with sand (3:1, *w*/*w*). The pots were kept under natural day/night conditions with photosynthetically active radiation (PAR) ∼640 µmol m^−2^ s^−1^ and average day/night temperature of 22/14 ± 3 °C and relative humidity 62–70% in a greenhouse of the Botany Department, Aligarh Muslim University, Aligarh, India.

The sulfur provided to plants was in the elemental form and at 200 mg kg^−1^ soil by applying 10 days before sowing, while 100 mM NaCl was given at the time of sowing and its application was made at alternate days for 15 days after sowing (DAS). The addition of 100 mM NaCl develops 10.0 dS m^−1^ salinity [86]. Our earlier findings have shown that 200 mg S kg^−1^ soil and 100 mg S kg^−1^ soil are excess-S and sufficient-S, respectively, and the excess-S increased photosynthesis and growth more than the sufficient-S in the presence of salt through GSH production [18]. The selection of NaCl concentration was based on our earlier research [18]. In the experimentation, the role of ethylene in mediating S-induced responses under salt stress was studied by using ethephon (2-chloroethyl phosphonic acid) as ethylene source. Ethephon at 200 µl L^−1^ was sprayed on the foliage at 20 DAS. The application of norbornadiene (NBD) was done at 100 μM concentration at 20 DAS. The surfactant Teepol (Azpack Ltd., Loughborough, UK) at 0.5% *v*/*v* was added to the foliar applications. The concentrations of ethephon and NBD were chosen based on previous research [21,25,44,59,60].

### 4.2. Biochemical Analyses

#### 4.2.1. Measurement of Na^+^ and Cl^−^ Content

The levels of Na^+^, Cl^−^, and S in roots and leaves were determined in the digested plant samples using Tri acid mixture (nitric acid, sulfuric acid and perchloric acid in the ratio of 10:5:4 *v*/*v*, respectively). The content of Na^+^ was estimated using flame photometer (Khera-391: Khera Instruments, New Delhi), whereas Cl^−^ content was determined by titration against 0.02 N silver nitrate solution using 5% K_2_CrO_4_ as indicator. The details are given in the Supplementary File S1.

#### 4.2.2. Measurement of H_2_O_2_ Content and Lipid Peroxidation

The content of H_2_O_2_ was determined following the method of Okuda et al. [87], while lipid peroxidation was determined by estimating TBARS content, according to Dhindsa et al. [88].

#### 4.2.3. Activity of ATP-Sulfurylase and Sulfur Content

The method of Lappartient and Touraine [89] was adopted for the measurement of ATP-S activity. The activity of ATP-S was assayed in leaves in vitro by measuring the molybdate-dependent formation of pyrophosphate.

The S content was determined by quadrupole inductively coupled plasma mass spectrometry (ICP-QMS) (model Elan DRC II; PerkinElmer SCIEX Inc., Shelton, CT, USA) [18]. High purity He and H_2_ were used to minimize the potential problems caused by unidentified reactive contaminant species.

### 4.3. Antioxidant Metabolism

#### Assay of Antioxidant Enzymes Activities

The methods of Nakano and Asada [90] and Foyer and Halliwell [91] were adopted for the measurement of APX and GR activity, respectively. For the assay of APX (EC 1.11.1.11) extraction buffer was supplemented with 2 mM AsA. DHAR (EC 1.8.5.1) activity was measured following the increase in absorbance at 265 nm because of the GSH dependent production of AsA as described by Foyer et al. [92]. MDHAR (EC 1.6.5.4) activity was measured adopting the method of Hossain et al. [93]. The details are given in the Supplementary File S1.

### 4.4. Glutathione and Ascorbate Pools

Reduced GSH and GSSG were assayed through the procedure described by Griffith [94]. Adopting Law et al. [95] dehydroascorbate (DHA) and reduced ascorbate (AsA) were determined through an enzymic recycling. In this, AsA was sequentially oxidized by 5,5-dithiobis-2-nitrobenzoic acid (DTNB) and then reduced by NADPH in the presence of GR.

### 4.5. ACS Activity and Ethylene Evolution

For the measurement of ACS (EC 4.4.1.14) activity, the method of Avni et al. [96] and Woeste et al. [97] was adopted. Ethylene was measured by placing 0.5 g of cut leaf material into 30 mL tubes containing moist paper to minimize evaporation from the tissue and stoppered with secure rubber caps and placed in light for 2 h under the same condition used for plant growth. The 1-mL gas sample of 1 mL was withdrawn from the tubes with a hypodermic syringe and assayed on a Nucon 5700 gas chromatograph (Nucon Engineers Private ltd., New Delhi, India) equipped with a 1.8-m Porapack^TM^ N (80–100 mesh) column (Sigma-Aldrich, St. Louis, MO, USA), a flame ionization detector and data station. Nitrogen was used as carrier gas. The flow rates of nitrogen, hydrogen, and oxygen were 30, 30, and 300 mL min^−1^, respectively. The detector was set at 150 ^◦^C. Ethylene was identified based on the retention time and quantified by comparison with peaks from standard ethylene concentration.

### 4.6. Abscisic Acid Determination

The content of ABA was determined by adopting the method of Hung and Kao [98] with slight modifications. Leaves were frozen with liquid nitrogen immediately and ground into fine powder. The powder was homogenized in the extraction solution (80% *v*/*v* methanol containing 2% *v*/*v* glacial acetic acid). The crude extract was centrifuged and passed through polyvinylpyrrolidone column and C18 cartridges to remove plant pigments and other non-polar compounds which could interfere in the immunoassay. The eluates were then concentrated to dryness by vacuum evaporation and resuspended in Tris-buffered saline. Afterwards, ABA was determined spectrophotometrically at 405 nm with an ABA immunoassay detection kit (model PGR-1; Sigma-Aldrich, St. Louis MO, USA).

### 4.7. Cytological and Histological Analysis

#### 4.7.1. Scanning Electron Microscopy

The leaf samples were prepared for scanning electron microscopy (SEM) by adopting the method of Daud et al. [99] with slight modifications. Fresh leaf samples were taken from the axillary positions (preferably, leaves were 4.0 × 4.0 cm in size) and air-dried in a desiccator. Subsequently, leaf samples were first fixed with 2.5% glutaraldehyde plus 2% paraformaldehyde in 0.1 M phosphate buffer (pH 7.0) in equal quantity for more than 4 h, and then washed three times with phosphate buffer for 15 min at each step. The samples were then post fixed with 1% osmium oxide in phosphate buffer (pH 7.0) for 1 h and washed three times with the same phosphate buffer for 15 min. The samples were dehydrated by a graded series of ethanol (50, 70, 80, 90, 95, and 100%) for about 15–20 min at each step, and transferred to the mixture of alcohol and isoamyl acetate (*v*/*v* = 1) for about 30 min. Then, the samples were transferred to pure iso-amyl acetate for 1 h and dehydrated with liquid CO_2_. The dehydrated specimen was coated with gold-palladium and observed under a Carl Zeiss EVO 40 scanning electron microscope (Zeiss, Aalen, Germany) at extra high tension and high voltage at 20 kV. The stomatal density was calculated from ten images obtained from different positions of each sample and circling 1-mm^2^ areas, according to Wang et al. [100].

#### 4.7.2. Transmission Electron Microscopy

Leaf tissues for chloroplast ultrastructure study were prepared for transmission electron microscopy (TEM) by adopting the method of Sandalio et al. [101] with slight modifications. The leaf samples were cut with a razor blade into 1 mm^2^-segments and fixed in 2.5% glutaraldehyde solution in 50 mM phosphate buffer (pH 6.8) for 2.5 h at room temperature. The leaf tissue was then post-fixed for 30 min in 1% osmium tetroxide in 50 mM sodium cacodylate buffer (pH 7.2) and dehydrated in ethanol graded series (30–100%, *v*/*v*). After dehydration in a graded series of ethanol, replaced to propylene oxide, and then the tissue was embedded in Spurr resin. Ultrathin sections were taken by using Leica EM UC6 ultramicrotome (Leica, Wetzlar, Germany). Sections were stained with uranyl acetate and lead citrate and examined by JEM-2100F field emission electron microscope a (Jeol Ltd.; Tokyo, Japan) accelerating voltage at 120 kV. The chloroplast ultrastructure (thylakoid membranes) was observed from TEM images. The number of thylakoids per granum and the number of grana stacks per chloroplast were estimated with the help of Image J open-source software from at least 10–15 images for each treatment.

#### 4.7.3. Confocal Microscopy

Young axillary leaves were picked and dried in a desiccator, and subsequently processed from the dorsal side to remove the epidermal layer and expose the stoma. The leaves were fine sectioned and mounted in a glycerol coverslip on glass slides. The samples were then analyzed under Olympus Fluoview TM-FV1000 (Olympus Life Sciences, Tokyo, Japan). Fluoview FV10 software, ver 1.7 (Olympus Life Sciences) was used to analyze and process the images.

### 4.8. Pigment Analysis and Leaf Gas Exchange

The content of chlorophyll (Chl) and carotenoids was measured with the method of Hiscox and Israelstam [102] by using dimethyl sulphoxide (DMSO) as an extraction medium and estimated and calculated by the method of Arnon [103]. Total Chl, Chl a, Chl b, and carotenoid levels were calculated according to the following equations:

Chl a = [(12.7 × OD_663_) − (2.69 × OD_645_)] × (V/(1000 × W))

Chl b = [(22.9 × OD_645_) − (4.68 × OD_663_)] × (V/(1000 × W))

Total Chl = [(20.2 × OD_645_) + (8.02 × OD_663_)] × (V/(1000 × W))

Carotenoids = [(7.6 × OD_480_) − (1.49 × OD_510_)] × (V/(1000 × W))

where V = volume of the extract, W = fresh mass of the tissue taken.

The relative amount of anthocyanins was estimated following the method of Mancinelli [104] with some modifications. Fresh leaf tissues (1 g) were grinded in acidified methanol (CH_3_OH: H_2_O: HCl, 79:20:1 *v*/*v*) and was centrifuged at 10,000 rpm for 5 min. The supernatant was collected and read spectrophotometrically at 530 and 657 nm, while the Chl and non-specific degradation products were corrected at 530 and 657 nm.

Infrared gas analyzer (model CID-340; Bio-Science, Camas, WA, USA) was used to measure gas exchange) on fully developed leaves randomly chosen for each treatment. The measurements were carried out between 10:00 and 11:00 a.m., at a flow rate of 500 µmol m^−2^ s^−1^. The same plants used for gas exchange measurements were chosen to measure chlorophyll fluorescence at 10:00–12:00 a.m. using a chlorophyll fluorometer (model Junior-PAM; Heinz Walz GmbH, Effeltrich, Germany). The maximal PSII photochemical efficiency (variable fluorescence to maximal fluorescence; *Fv*/*Fm*) was determined on the second leaf from the top of the plant between 10:00 and 11:00 a.m., according to Khan and Khan [105].

The leaf area was measured with a leaf area meter (model LA 211; Systronics, New Delhi, India), while plant fresh weight trough a digital scale (Sartorius, Göttingen, Germany).

### 4.9. Water Use Efficiency and Stomatal Aperture

Water use efficiency was determined by calculating the ratio of net photosynthesis by stomatal conductance [106]. Stomatal apertures were examined by confocal microscopy, as described above.

### 4.10. Statistical Analysis

The treatments of the experiments were arranged in completely randomized block design, and each treatment had four replicates (*n* = 4). All measurements were done at 30 DAS. The data were analyzed statistically using analysis of variance (ANOVA) by SPSS17.0 for Windows and presented as a treatment mean ± SE (*n* = 4). Least significant difference (LSD) was calculated for the significant data at *p* < 0.05.

## 5. Conclusions

Salinity stress is detrimental to crop growth and development and needs attention for avoidance/tolerance mechanism. The modulation of hormones/nutrients provides a suitable strategy for the sustainable development of plants under salinity stress. The results showed that both ethephon or/and S increased the stomatal and photosynthetic response of plants with or without salt stress. S-assimilation is an important strategy to increase plant antioxidative property to deal with salt stress and an increase in photosynthetic efficiency. The regulatory interaction between ethephon and S signal can control plant growth, development, and metabolism under salt stress conditions through both stomatal and non-stomatal regulation. The ABA-mediated stomatal closure with increased H_2_O_2_ content was reversed by ethylene plus S application. Ethephon and S also decreased H_2_O_2_ accumulation by increasing the components of AsA-GSH cycle, and reduced ABA content, causing the inhibition of stomatal closure and the maintenance of photosynthesis under salt stress.

The involvement of ethylene in the regulation of the antioxidant system, and on the structure and function of the photosynthetic apparatus was ascertained with the inhibition of ethylene action by NBD. Ethylene was found to increase ABA content under salt stress, which was suggestive of ethylene action in the reduction of ABA content and ABA-mediated stomatal closure. Therefore, the coordinated action of ethephon and S efficiently reduced salinity-induced oxidative stress via regulating the antioxidant system involving components of AsA-GSH cycle and ABA to inhibit stomatal closure. The effects of ethephon and sulfur on photosynthesis were through regulation of stomatal conductance and the availability of intercellular CO_2_ concentration.

The results suggest that, in *B. juncea*, ethephon and S can modulate antioxidant system and ABA accumulation in guard cells, controlling stomatal conductance and the structure and efficiency of the photosynthetic apparatus in plants under salt stress. In this process, ethylene has a key role. However, ethylene is not independent in its action and, besides ABA, it influences various other phytohormones. Thus, we can conclude that, although we have focused on ethylene and ABA and their role in in stomatal regulation under salt stress, the possibility of interaction between ABA, NO, H_2_O_2_, Ca, MeJA, auxin, and various other signaling compounds cannot be ignored and still needs to be explored.

## Figures and Tables

**Table 1:** Content of Na^+^ and Cl^−^ in roots and leaves of mustard plants at 30 days after sowing. Plants were treated with 200 µL L^−1^ ethephon and/or 200 mg S kg^−1^ soil (S) in presence or absence of 100 mM NaCl. Data are presented as means ± SE (*n* = 4). Data followed by same letter are not significantly different by LSD test at *p* < 0.05.

Treatments	Roots		Leaves	
	Na^+^	Cl^−^	Na^+^	Cl^−^
	(mg g^−1^ Leaf Fresh Weight)			
Control	9.20 ± 1.03 e	7.70 ± 0.34 e	8.44 ± 0.70 e	6.81 ± 0.58 e
NaCl	27.31 ± 0.41 a	17.90 ± 0.42 a	22.36 ± 0.99 a	15.64 ± 0.76 a
Ethephon (E)	7.74 ± 0.66 f	5.50 ± 0.29 f	6.95 ± 0.63 f	5.22 ± 0.62 f
Sulfur (S)	7.66 ± 0.63 f	5.21 ± 0.29 f	6.64 ± 0.60 f	5.03 ± 0.55 f
E + S	6.61 ± 0.14 g	4.12 ± 0.09 g	4.89 ± 0.11 g	4.17 ± 0.09 g
E + NaCl	15.90 ± 0.73 b	11.90 ± 0.59 b	15.30 ± 0.99 b	11.20 ± 0.65 b
S + NaCl	14.55 ± 0.70 c	10.90 ± 0.48 c	12.72 ± 0.75 c	9.51 ± 0.73 c
E + S + NaCl	10.75 ± 1.07 d	8.70 ± 0.28 d	11.00 ± 0.89 d	8.30 ± 0.65 d

**Table 2:** Content of H_2_O_2_, TBARS, activity of APX, GR, DHAR and MDHAR and GSH content and redox state (GSH/GSSG) in mustard leaves at 30 days after sowing. Plants were treated with 200 µL L^−1^ ethephon and/or 200 mg S kg^−1^ soil (S) in presence or absence of 100 mM NaCl. Data are presented as means ± SE (*n* = 4). Data followed by same letter are not significantly different by LSD test at *p* < 0.05. APX, ascorbate peroxidase; GSH, reduced glutathione; DHAR, dehydroascorbate reductase; GR, glutathione reductase; H_2_O_2_ hydrogen peroxide; MDHAR, monodehydroascorbate reductase; GSSG, oxidized glutathione; TBARS, thiobarbituric acid reactive substances.

Treatments	H_2_O_2_	TBARS	APX	GR	DHAR	MDHAR	GSH	GSSG	Redox State
	(nmol g^−1^ Leaf Fresh Weight)		(U mg^−1^ Protein min^−1^)				(nmol g^−1^ Leaf Fresh Weight)		
Control	16.1 ± 0.9 b	4.20 ± 0.22 b	1.22 ± 0.05 g	0.204 ± 0.007 g	90.1± 3.7 g	60 ± 3 g	58.2 ± 3.3 g	2.9 ± 0.2 c	21.0 ± 0.6 f
NaCl	32.4 ± 1.0 a	8.30 ± 0.23 a	1.69 ± 0.06 f	0.248 ± 0.009 f	124.0 ± 4.1 f	110 ± 3 f	71.0 ± 3.6 f	8.5 ± 0.6 a	8.4 ± 0.6 g
Ethephon (E)	8.7 ± 0.7 e	2.70 ± 0.19 e	3.02 ± 0.09 c	0.363 ± 0.012 c	162.0 ± 4.7 c	144 ± 4 c	98.6 ± 2.6 c	3.3 ± 0.2 c	30.0 ± 0.7 c
Sulfur (S)	8.1 ± 0.8 e	2.50 ± 0.18 e	3.09 ± 0.09 c	0.370 ± 0.013 c	165.0 ± 4.8 c	149 ± 4 c	101.7 ± 3.9 c	3.2 ± 0.7 c	31.1 ± 0.7 c
E + S	4.1 ± 0.5 g	1.64 ± 0.14 g	3.90 ± 0.11 a	0.435 ± 0.014 a	196.0 ± 5.1 a	172 ± 5 a	115.4 ± 3.5 a	3.0 ± 1.0 c	38.4 ± 1.0 a
E + NaCl	13.2 ± 0.8 c	3.70 ± 0.20 c	2.12 ± 0.07 e	0.311 ± 0.010 e	135.0 ± 4.5 e	121 ± 4 e	80.4 ± 2.5 e	3.8 ± 0.5 b	21.3 ± 0.7 e
S + NaCl	11.6 ± 0.6 d	3.20 ± 0.19 d	2.43 ± 0.08 d	0.341 ± 0.011 d	147.0 ± 4.7 d	133 ± 4 d	88.0 ± 2.5 d	3.7 ± 0.6 b	24.1 ± 0.7 d
E + S + NaCl	5.7 ± 0.7 f	2.00 ± 0.17 f	3.40 ± 0.10 b	0.400 ± 0.012 b	180.0 ± 5.0 b	160 ± 5 b	110.3 ± 3.2 b	3.1 ± 0.4 c	36.1 ± 1.0 b

**Table 3:** Activity of ATP-sulfurylase (ATP-S), and content of sulfur (S), ascorbate (AsA), and dehydroascorbate (DHA) in mustard leaves at 30 days after sowing. Plants were treated with 200 µL L^−1^ ethephon and/or 200 mg S kg^−1^ soil (S) in presence or absence of 100 mM NaCl. Data are presented as means ± SE (*n* = 4). Data followed by same letter are not significantly different by LSD test at *p* < 0.05.

Treatments	ATP-S Activity	S	AsA	DHA
	(µmol g^−1^ Protein s^−1^)	(mg g^−1^ Fresh Weight)	(nmol g^−1^ Fresh Weight)	(nmol g^−1^ Fresh Weight)
Control	1.24 ± 0.06 g	5.0 ± 0.2 f	22.31 ± 0.77 g	05.28 ± 0.32 g
NaCl	1.44 ± 0.04 f	4.1 ± 0.1 g	27.50 ± 0.98 f	09.20 ± 0.33 f
Ethephon (E)	2.31 ± 0.07 c	7.7 ± 0.3 c	37.62 ± 1.10 c	15.40 ± 0.39 d
Sulfur (S)	2.36 ± 0.07 c	7.9 ± 0.3 c	38.28 ± 1.13 c	15.84 ± 0.41 c
E + S	2.87 ± 0.08 a	9.1 ± 0.3 a	46.20 ± 1.34 a	18.48 ± 0.41 a
E + NaCl	1.88 ± 0.06 e	6.4 ± 0.3 e	31.51 ± 1.03 e	13.11 ± 0.33 e
S + NaCl	2.15 ± 0.06 d	7.0 ± 0.3 d	34.27 ± 1.09 d	14.26 ± 0.37 d
E + S + NaCl	2.54 ± 0.08 b	8.6 ± 0.3 b	43.24 ± 1.28 b	17.71 ± 0.35 b

**Table 4:** Content of chlorophyll a (Chl a), Chl b, total Chl, carotenoids and relative amounts of anthocyanins of mustard leaves at 30 days after sowing. Plants were treated with 200 µL L^−1^ ethephon (E) and/or 200 mg S kg^−1^ soil in presence or absence of 100 mM NaCl. Data are presented as means ± SE (*n* = 4). Data followed by same letter are not significantly different by LSD test at *p* < 0.05.

Treatments	Chl a	Chl b	Total Chl	Carotenoids	Anthocyanins
	(mg g^−1^ Leaf Fresh Weight)				
Control	1.31 ± 0.05 f	0.62 ± 0.02 f	2.00 ± 0.07 f	0.57 ± 0.02 f	0.61 ± 0.03 g
NaCl	1.09 ± 0.05 g	0.41 ± 0.01 g	1.56 ± 0.06 g	0.46 ± 0.02 g	1.33 ± 0.05 f
Ethephon (E)	1.58 ± 0.06 c	0.82 ± 0.02 c	2.40 ± 0.08 c	0.69 ± 0.03 c	1.61 ± 0.06 c
Sulfur (S)	1.61 ± 0.06 c	0.84 ± 0.03 c	2.43 ± 0.08 c	0.71 ± 0.03 c	1.64 ± 0.06 c
E + S	1.81 ± 0.07 a	1.05 ± 0.03 a	2.86 ± 0.09 a	0.83 ± 0.04 a	1.82 ± 0.07 a
E + NaCl	1.41 ± 0.06 e	0.68 ± 0.02 e	2.10 ± 0.07 e	0.60 ± 0.03 e	1.46 ± 0.06 e
S + NaCl	1.49 ± 0.04 d	0.75 ± 0.02 d	2.24 ± 0.08 d	0.66 ± 0.03 d	1.54 ± 0.06 d
E + S + NaCl	1.70 ± 0.07 b	0.94 ± 0.03 b	2.64 ± 0.09 b	0.78 ± 0.03 b	1.73 ± 0.06 b

**Table 5:** Maximal PSII photochemical efficiency, net photosynthesis, intercellular CO_2_ concentration, stomatal conductance, water use efficiency, leaf area and plant fresh weight of mustard leaves at 30 days after sowing. Plants were treated with 200 µL L^−1^ ethephon (E) and/or 200 mg S kg^−1^ soil (S) in the presence or absence of 100 mM NaCl. Data are presented as means ± SE (*n* = 4). Data followed by the same letter are not significantly different by LSD test at (*p* < 0.05).

Treatments	Maximal PSII Photochemical Efficiency	Net Photosynthesis	Intercellular CO_2_ Concentration	Stomatal Conductance	Water Use Efficiency	Leaf Area	Plant Fresh Weight
		(µmol CO_2_ m^−2^ s^−1^)	(µmol CO_2_ mol^−1^)	(mmol H_2_O m^−2^ s^−1^)	(µmol mol^−1^)	(cm^2^ Plant^−1^)	(g Plant^−1^)
Control	0.78 ± 0.02 f	13.7 ± 0.6 f	257 ± 10 f	377 ± 11 f	37.1 ± 1.5 f	117.0 ± 3.9 f	2.15 ± 0.06 f
NaCl	0.65 ± 0.01 g	8.1 ± 0.6 g	176 ± 7 g	298 ± 9 g	29.4 ± 1.3 g	59.4 ± 2.5 g	1.01 ± 0.05 g
Ethephon (E)	0.90 ± 0.02 c	19.3 ± 0.7 c	357 ± 14 c	481 ± 19 c	49.1 ± 2.3 c	164.8 ± 5.1 c	2.94 ± 0.09 c
Sulfur (S)	0.91 ± 0.02 c	19.6 ± 0.7 c	365 ± 15 c	487 ± 19 c	50.0 ± 2.3 c	168.7 ± 5.3 c	3.00 ± 0.08 c
E + S	0.99 ± 0.02 a	22.2 ± 0.7 a	433 ± 16 a	590 ± 22 a	60.0 ± 2.4 a	207.0 ± 5.8 a	3.81 ± 0.09 a
E +NaCl	0.83 ± 0.02 e	16.5 ± 0.8 e	300 ± 12 e	408 ± 15 e	40.8 ± 1.6 e	138.7 ± 4.7 e	2.56 ± 0.07 e
S + NaCl	0.87 ± 0.02 d	18.2 ± 0.6 d	329 ± 13 d	449 ± 17 d	43.8 ± 1.7 d	155.7 ± 4.8 d	2.78 ± 0.08 d
E + S + NaCl	0.96 ± 0.02 b	21.4 ± 0.7 b	398 ± 15 b	550 ± 21 b	56.0 ± 2.5 b	185.0 ± 5.5 b	3.54 ± 0.09 b

**Table 6:** Content of ABA, H_2_O_2_, net photosynthesis, and stomatal conductance in mustard leaves at 30 days after sowing. Plants were treated with 200 µL L^−1^ ethephon (E) and 200 mg S kg^−1^ soil (S) in presence or absence of 100 mM NaCl. The treatment with 100 μM norbornadiene (NBD) was applied either alone or with combination of 200 µL L^−1^ ethephon (E) and 200 mg S kg^−1^ soil (S) with 100 mM NaCl. Data are presented as means ± SE (*n* = 4). Data followed by same letter are not significantly different by LSD test at (*p* < 0.05). E, ethephon; NBD, norbornadiene; S, sulfur.

Treatments	ABA	H_2_O_2_	Net Photosynthesis	Stomatal Conductance
	(pmol g^−1^ Fresh Weight)	(nmol g^−1^ Fresh Weight)	(µmol CO_2_ m^−2^ s^−1^)	(mmol H_2_O m^−2^ s^−1^)
Control	70.0 ± 5.2 e	16.4 ± 0.9 d	13.0 ± 0.6 c	373 ± 11 c
NaCl	250.0 ± 8.8 a	31.9 ± 1.1 a	7.5 ± 0.6 f	275 ± 9 e
E + S	54.7 ± 4.4 f	4.3 ± 0.6 f	27.6 ± 1.1 a	582 ± 22 a
E + S + NaCl	101.0 ± 8.3 d	8.3 ± 0.3 e	24.0 ± 0.8 b	540 ± 20 b
NBD	71.2 ± 6.0 e	22.0 ± 0.8 c	12.1 ± 0.7 d	351 ± 12 d
E + S + NaCl + NBD	230.4 ± 8.9 b	28.0 ± 0.9 b	8.1 ± 0.6 e	255 ± 10 f

## Data Availability

The data presented in this study are available in the graphs and tables provided in the manuscript.

## Supplementary Materials

The following are available online at https://www.mdpi.com/2223-7747/10/1/180/s1.
