# Supplementary material for: Ethylene and Sulfur Coordinately Modulate the Antioxidant System and ABA Accumulation in Mustard Plants under Salt Stress
Source: Plants (Basel). 2021 Jan 19;10(1):180. doi: 10.3390/plants10010180 (PMC7835815; doi:10.3390/plants10010180)
Supplement: Supplementary file 1 [file plants-10-00180-s001.pdf]

Supplementary File S1

Measurement of Na^+ and Cl^-content ions

The content of Na^+ and Cl^- in leaf and root were determined in the digested plant samples using Tri acid mixture (TAM; a mixture of nitric acid, sulfuric acid and perchloric acid in the ratio of 10:5:4). Extraction of the content of ions was done by boiling for 30 mins, in distilled water. The extract was filtered to measure specific ions. The content of Na^+ was estimated using flame photometer (Khera-391: Khera Instruments, New Delhi), whereas Cl^- content was determined by titration against 0.02 N silver nitrate solution using 5% K_2CrO_4 as indicator.

Assay of antioxidant enzymes activities

The method of Nakano and Asada (1981) and Foyer and Halliwell (1976) with slight modifications, were adopted for the measurement of APX, and GR activity, respectively. Fresh leaf tissues (200 mg) were homogenized in chilled mortar and pestle with an extraction buffer containing 0.05% (v/v) Triton X-100 and 1% (w/v) polyvinylpyrrolidone in 100 mM potassium-phosphate buffer (pH 7.0). At 4 ˚C, the homogenate was centrifuged at 15,000 × g for 20 min. The supernatant obtained after centrifugation was used for the assay of GR (EC; 1.6.4.2). For the assay of APX (EC; 1.11.1.11) extraction buffer was supplemented with 2mM AsA. The details of procedure have been described earlier in our studies (Fatma et al., 2014). DHAR (EC; 1.8.5.1) activity was measured following the increase in absorbance at 265 nm due to the GSH dependent production of reduced ascorbate (AsA) as described by Foyer et al. (1989). The reaction mixture contained potassium phosphate buffer (0.1 M, pH 6.2), 2 mM GSH, and 50-100 g of proteins (Bradford, 1976). The reaction was started upon addition of 1 mM DHA. One enzyme unit was equivalent to nmol AsA/(g dry weight min).

MDHAR (EC, 1.6.5.4) activity was measured adopting the method of Hossain et al. (1984). The reaction mixture was prepared by adding 50 mM Tris-HCl buffer (pH 7.5), 0.2 mM NADPH, 2.5 mM AsA, 0.5 unit of ascorbate oxidase and enzyme solution and the final volume was made 700 μL. The reaction was started by the addition of ascorbate oxidase and the activity was calculated from the change in absorbance at 340 nm for 1 min using an extinction coefficient of 6.2 mM^{-1} cm^{-1}

Content of GSH, redox state, reduced and oxidized ascorbate contents

Adopting the procedure of Griffith (1980) reduced GSH was assayed through an enzymic recycling. In this, sequentially oxidized by 5,5-dithiobis-2-nitrobenzoic acid (DTNB) and reduced by NADPH in the presence of GR. The details for the determination of GSH and redox state have been elaborated in Fatma et al. (2014).

Adopting Law et al. (1983) oxidized (DHA) ascorbate were determined with some modifications. Fresh leaves were homogenized in 2 mL of potassium phosphate buffer (100 mM, pH 7.0) containing 1 mM EDTA and centrifuged at 10000× g for 10 min. To 1 mL of the supernatant, 0.5 ml of 10% (w/v) TCA was added, thoroughly mixed, and incubated for 5 min at 4˚C. The 1.5 mL of the above solution and 0.5 ml of NaOH (0.1 M) were thoroughly mixed and centrifuged at 5000 × g for 10 min at 20˚C. The aliquot thus obtained was equally distributed into two separate microfuge tubes (750 μL each). For the estimation of AsA, 200 μL of potassium phosphate buffer (150 mM, pH 7.4) was added to 750 μL of the aliquot, while for DHA estimation, 750 μL of the aliquot was added to100 μL of DTT followed by vortex-mixing, incubation for 15 min at 20˚C, and addition of 100 μL of 0.5% (w/v) NEM. Then, both microfuge tubes were incubated for 30 sec at room temperature. To each sample tube, 400 μL of 10% (w/v) TCA, 400 μL of H_3PO_4, 400 μL of 4% (w/v) bipyridyl dye (N′, N-dimethyl bipyridyl), and 200 μL of 3% (w/v) $FeCl_3$were added and thoroughly mixed. The absorbance was recorded at 525 nm after incubation for 1 h at 37˚C. A standard curve in the range of 5-55 nmol of AsA was used for calibration. For DHA, standard curve covering a range of 1-5 nmol was used.
